# Microbial Population Dynamics in Model Sewage Treatment Plants and the Fate and Effect of Gold Nanoparticles

**DOI:** 10.3390/toxics9030054

**Published:** 2021-03-10

**Authors:** Karsten Schlich, Cecilia Díaz, Benjamin Gomez Pizarro, Burkhard Knopf, Ruben Schlinkert, Franziska Frederike Wege, Anne Jurack, Kerstin Hund-Rinke

**Affiliations:** 1 Department of Ecotoxicology, Fraunhofer Institute for Molecular Biology and Applied Ecology, Auf dem Aberg 1, 57392 Schmallenberg, Germany; cecilia.andrea.diaz.navarrete@ime.fraunhofer.de (C.D.); b.gmezpizarro@uandresbello.edu (B.G.P.); burkhard.knopf@ime.fraunhofer.de (B.K.); ruben.schlinkert@ime.fraunhofer.de (R.S.); franziska-frederike.wege@ime.fraunhofer.de (F.F.W.); a_jura01@uni-muenster.de (A.J.); kerstin.hund-rinke@ime.fraunhofer.de (K.H.-R.); 2 Facultad de Ciencias de la Vida, Universidad Andrés Bello, Echaurren 281, Santiago 8370146 RM, Chile; 3 Institute for Plant Biology and Biotechnology, University of Münster, Schlossplatz 7–8, 48143 Münster, Germany

**Keywords:** nanotoxicology, sewage treatment plant, microbial diversity, nanoparticles, fate

## Abstract

Adequate functioning of a sewage treatment plant (STP) is essential to protect the downstream aquatic environment (ECHA 2017), and information on the degradability of chemicals and their toxicity to activated sludge microorganisms is required. An environmental realistic higher tier test is a STP simulation test as described in OECD 303A (2001) which for nanoparticles can also be used to study their sorption behavior to activated sludge. However, information is limited on the influence of synthetic sewage on the microbial community of the activated sludge. A modified community can result in modifications of the sludge floccules affecting the sorption behavior. The main objective of our study was to show whether a representative microbial diversity remains under standardized test conditions as described in OECD 303A (2001) using synthetic sewage as influent. Furthermore, we investigated whether just considering the functional properties of a STP (elimination of dissolved organic carbon; nitrification), is sufficient for an assessment of gold nanoparticles (AuNPs) or whether the influence on microbial diversity also needs to be considered. AuNPs were used as a case study due to their rising medical applications and therefore increasing probability to reach the sewer and STP. The results can provide significant input for the interpretation of results from the regulatory point of view. To deliver these objectives, the general changes of the microbial population in activated sludge and its influence on the degradation activity (dissolved organic carbon (DOC) and inorganic nitrogen) using freshly collected sludge from the municipal STP in an artificial test system as a model STP in accordance with OECD 303A (2001) were assessed. Additionally, we evaluated the potential impact of AuNPs and its dispersant on the microbial composition and the overall impact on the function of the STP in terms of DOC degradation and nitrogen removal to observe if an assessment based on functional properties is sufficient. The bacteria composition in our study, evaluated at a class level, revealed commonly described environmental bacteria. Proteobacteria (β, α, δ) accounted for more than 50% but also nitrifying bacteria as *Nitrospira* were present. Our results show that mainly within the first 7 days of an acclimatization phase by addition of synthetic sewage, the bacterial community changed. Even though AuNPs can have antibacterial properties, no adverse effects on the function and structure of the microorganisms in the STP could be detected at concentrations of increased modeled PEC values by a factor of about 10,000. Complementary to other metallic nanomaterials, gold nanomaterials also sorb to a large extent to the activated sludge. If activated sludge is used as fertilizer on agricultural land, gold nanoparticles can be introduced into soils. In this case, the effect on soil (micro)organisms must be investigated more closely, also taking into account the structural diversity.

## 1. Introduction

Adequate functioning of a sewage treatment plant (STP) is essential to protect the downstream aquatic environment and to minimize operational costs [1]. Therefore, for the Registration, Evaluation, Authorisation and Restriction of Chemicals (REACH) managed by the European Chemicals Agency (ECHA), information on degradability of chemicals and their (eco-)toxicity on activated sludge microorganisms is required, among others. The most environmental realistic higher tier test is a STP simulation test such as described in OECD Guideline 303A [2].

The OECD 303A (2001) [2] was developed for the testing of water-soluble and non-volatile chemicals. In a continuously operating system comprising an aerobic chamber and a settling vessel, activated sludge derived from a municipal STP is fed with synthetic sewage. According to the OECD 303A (2001) [2], the influent and effluent dissolved organic carbon (DOC) is measured providing information on the removal of a test substance including degradation and adsorption. The anaerobic microbial reduction of nitrate (denitrification) to decrease the nitrate concentration is not taken into account. Based on the obtained results, predicted non-effect concentrations (PNEC) values can be calculated using parameters such as biological oxygen demand (BOD), chemical oxygen demand (COD), or nitrogen removal.

The test design is considered to represent a realistic approximation to actual conditions in full-scale STPs [1]. However, the composition of the synthetic sewage with peptone, meat extract, urea, and various salts is rather simple compared to real sewage. It is well known that the composition of the influent in STPs affects the composition of the microbial population and the degradation spectrum for organic substances [3,4,5,6]. Therefore, changes in the microbial population of the activated sludge due to the incubation under laboratory conditions have to be expected. The addition of the test substance starts when the system has been stabilized and is removing DOC efficiently. Finally, the addition of the test substance might also result in additional modifications of the microbial population. According to the guideline, modifications of the microbial community are not considered.

In Annex 5 of OECD 303A (2001) [2], information on the testing of poorly water-soluble test substances is provided, and the test approach has also been found to be suitable for the investigation of nanomaterials, in particular for assessing the distribution of the particles in STPs [7]. For nanomaterials, it is explicitly stated that the nitrifying microorganisms, besides the organic carbon degrading microorganisms, must be considered. This is because nitrifiers are very sensitive and potential toxic effects can already be detected at test concentrations which do not affect the degradation potential [8]. Currently, there are several molecular tools to investigate changes in microbial communities, which allow studying single species as well as the whole community, e.g., polymerase chain reaction (PCR), denaturing gradient gel electrophoresis (DGGE), automated ribosomal intergenic spacer analysis (ARISA)-PCR, or next-generation sequencing (NGS). The use of the NGS technology targeting the bacterial and archaeal 16S rRNA genes allows tracking changes in the overall community and also provides information to particular groups of interest such as nitrifiers.

In the current period during the Covid-19 pandemic, nanoparticles (NPs) can offer alternative methods to classical disinfection protocols used in healthcare settings, thanks to their intrinsic antipathogenic properties and/or their ability to inactivate viruses, bacteria, fungi, or yeasts either photothermally or via photocatalysis-induced reactive oxygen species (ROS) generation [9]. Among others, gold nanoparticles (AuNPs) are used as antimicrobial agents and for drug delivery. Due to their use, they will enter the environment by, e.g., excretion by the patients, and thereby will be released into the sewer system and consequently into the STP. Ecotoxicological impacts of AuNPs can range from no or minimal effect [10,11,12] to significant effects [13,14] in the aquatic environment. Observing effects on microorganisms in STPs, no inhibition of the ammonium oxidizing bacteria (AOB) biodiversity, abundance, and the ammonium oxidation was reported [15,16].

The main objective of our study was to show whether a representative microbial diversity persists under standardized test conditions as described in OECD 303A (2001) [2] using synthetic activated sludge as influent. Furthermore, we investigated whether only considering the functional properties of a STP, such as DOC elimination, is sufficient for an assessment of AuNPs ecotoxicity or whether the influence on microbial diversity also needs to be considered. The results can provide significant input for the interpretation of results from the regulatory point of view.

To deliver these objectives, the general changes of the microbial population in activated sludge as well as the influence on the degradation activity (DOC and nitrogen) were assessed using freshly collected sludge from the municipal STP in an artificial STP test system following the OECD 303A (2001) [2]. Additionally, we evaluated the potential impact of AuNPs on the microbial composition and the overall impact on the function of the STP in terms of DOC degradation and nitrogen removal to observe if an assessment based on functional properties is sufficient. The NPs were available as dispersion. Therefore, additionally the effect of the pure dispersant was investigated.

## 2. Materials and Methods

### 2.1. Gold Nanoparticles

The properties of the AuNPs and their respective dispersant are briefly described in Table 1, and both were provided by Colorobbia Consulting srl (Florence, Italy). The AuNPs were available in an aqueous solution of sodium citrate (1 mM) containing Polyvinylpyrrolidon (PVP) as stabilizer (0.27 ± 0.5 [%wt]).

### 2.2. Activated Sludge

Activated sludge was taken from the municipal STP of Schmallenberg, Germany, and transported to the test facilities. The sludge fulfilled the criteria as stated in the German Sewage Sludge Ordinance [17] regarding the permitted heavy metal thresholds. The dry matter (dm) content of the activated sludge was determined using a moisture analyzer (Mettler Toledo, HC 103). Afterward, the activated sludge was poured equally into the nitrification and denitrification tanks to achieve a dry matter content of 2.5 g dm sludge/L after addition of tap water to a final volume of 10 L.

### 2.3. Model Sewage Treatment Plant

The effect of AuNPs and the dispersant was evaluated using the model STPs. In addition, the fate of the AuNPs was observed. The effect was investigated by determination of the degradation activity of the microorganisms in the activated sludge using the DOC values measured in influent and effluent of the respective measurement points and by the changes in the community assessed by DNA sequencing.

The STP simulation was carried out according OECD Guideline 303A (2001) [2] using laboratory STPs (behrost^®^ laboratory wastewater treatment plant KLD 4N, Düsseldorf, Germany) equipped with a denitrification tank (anaerobic), a nitrification tank (aerobic), and a settling tank. The total volume of the laboratory STP was 10 L, using 4.5 L for the denitrification, 3.5 L for the nitrification, and 2 L for the settling tank. Synthetic sewage was continuously dosed into the STPs denitrification vessel. For suitable denitrification conditions, the settled activated sludge was periodically pumped from the nitrification vessel into the denitrification vessel.

Synthetic sewage was prepared following OECD 303A [2] containing 160 mg peptone, 110 mg meat extract, 30 mg urea, 28 mg K_2_HPO_4_, 7 mg NaCl, 4 mg CaCl_2_ × 2 H_2_O, and 2 mg MgSO_4_ × 7 H_2_O per liter of deionized water. The synthetic sewage was concentrated ten times and stored in the refrigerator at 4 °C. Tap water from a 200 L storage container was fed into the tube system. Using a Y-connector, synthetic sewage, concentrated 10-fold, was added to the tube system and mixed to achieve a dissolved organic carbon concentration of 150 ± 20 mg/L. The flow rate was adjusted to 750 mL/h, which led to a retention time of 6 hours in the nitrification tank of the STP. The flow rate was checked at least every three days. The amount of DOC in the influent and effluent of the STP was measured every second day within the complete test period (18 days) with a TOC-VCPH Total Organic Carbon Analyzer (Shimadzu, Duisburg, Germany). Based on the elimination of the DOC, the degradation rate was determined.

Due to the limited amount of AuNPs, we could not perform studies on the stability of AuNPs in a dispersion which could be added continuously to the STPs. Therefore, it was decided to add the AuNPs directly into the denitrification tank five times daily. AuNPs were added from day 0 to day 9 (10 days total). Over the whole experiment, the AuNP stock and its dispersant were continuously kept homogeneous using an overhead shaker (Reax 20, Heidolph Instruments GmbH & Co. KG, Schwabach, Germany). For addition of the AuNPs or the dispersant into the STP, 950 µL was mixed with 75 mL of synthetic sewage. The dispersion was homogenized for 5 min in an ultrasonic bath (Sonorex, Bandelin electronic GmbH & Co. KG, Berlin, Germany) and then added directly into the denitrification tank. For the control, the same procedure was applied using 75 mL synthetic sewage. This application rate led to a final concentration of AuNPs in the activated sludge of ~7 g/kg. The concentration used for the experiments therefore exceeded modeled PEC values [18] by a factor of approximately 10,000 and represents a worst-case scenario.

Various water parameters (pH, dry matter content, nitrate, nitrite, and ammonium) were documented during the experiments to prove that the STP simulation was in line with the OECD 303A and to show the comparability between the different replicates. The dry matter content in the nitrification and denitrification tank was measured every three days. In addition, the pH was measured periodically in the denitrification and nitrification tank and the influent and effluent. The nitrate, nitrite, and ammonium concentrations in the effluent were determined three times a week using a Nanocolor^®^ 500 (MACHEREY-NAGEL Inc., Düren, Germany). Over the entire test period, the oxygen content in the nitrification tank was regulated to be between 2.0 and 3.5 mg O_2_/L.

To determine the general changes of the microbial population in the STP and those induced due to the addition of AuNPs or the dispersant, samples of fresh sludge were taken from the nitrification vessel for DNA analysis. The first sample was taken immediately after the activated sludge was received from the municipal sewage treatment plant in order to have a starting point (day −8). A second sample in each experiment was taken during the acclimatization phase (day −4) to show the immediate effect of standardized environmental conditions, e.g., using synthetic sewage. Afterward, samples were taken at test start (day 0), 3 days after the addition of dispersant or AuNPs, and after 9 days, when the STP simulation was terminated. At each sampling, one sample per replicate was taken (*n* = 2) per treatment (control, dispersant, or AuNPs).

Given the STP capacity (four STPs available), the experiment was conducted in two separated experiments to (i) evaluate general changes in microbial diversity during the time course of the experiment and the effect of the dispersant, and (ii) to observe if the STPs function is sufficient to assess the impact of AuNPs on the STP.

For each experiment, two STP replicates were conducted per treatment. Both experiments provided information on the general changes of microbial diversity in sludge and on the repeatability of tests with natural activated sludge. The second experiment additionally showed the potential effect of AuNP on the STP function and the microbial population in sludge.

In the first experiment, we checked whether the dispersant of the AuNP influenced the microbial diversity by comparing it with the microbial diversity of a control treatment, fed only with synthetic sewage as described in OECD 303A [2]. The focus of the second experiment was on the fate and effect of AuNPs in the STP. Therefore, the test design included two replicates receiving dispersed AuNPs and two replicates receiving only the dispersant of the AuNPs (without particles). In this case, the treatment with the dispersant was used as control.

**Figure 1:**
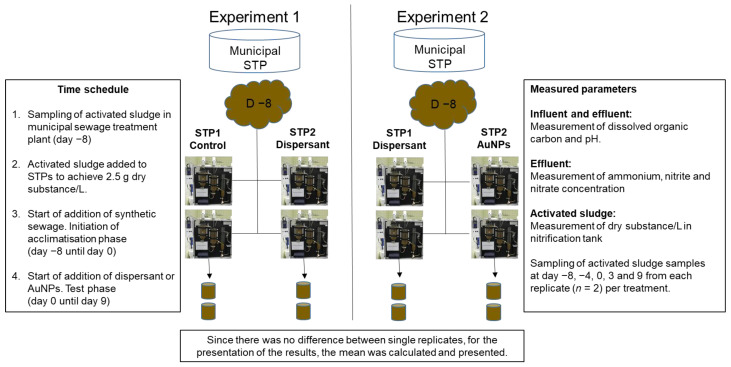
Schematic representation of the temporal sequence and the measurements carried out in the two experiments (experiment 1: control vs. dispersant and experiment 2: dispersant vs. AuNPs).

A scheme of the experimental design is presented in Figure 1.

### 2.4. Microbial Community Analysis

Activated sludge samples were collected during the whole experiment as described above. Total genomic DNA (gDNA) was extracted from each sample, in duplicate, using the DNeasy^®^ PowerSoil^®^ Kit (Qiagen, Hilden, Germany) according to the instructions of the provider. The concentration and purity of the extracts was measured using a Nano Drop™ 2000 (Thermo Scientific, Waltham, MA, USA). The DNA of the replicates was pooled and the concentration adjusted to 20 ng/µL before sequencing.

16S rDNA next-generation sequencing library preparations and Illumina MiSeq sequencing were conducted at GENEWIZ, Inc. (South Plainfield, NJ, USA). DNA samples were quantified using a Qubit 2.0 Fluorometer (Invitrogen, Carlsbad, CA, USA) and DNA quality was checked on a 0.6% agarose gel. Sequencing library was constructed using a MetaVx™ 16s rDNA Library Preparation kit (GENEWIZ, Inc., South Plainfield, NJ, USA). Briefly, the DNA was used to generate amplicons that cover V3 and V4 hypervariable regions of bacterial and archaeal 16S rDNA. Indexed adapters were added to the ends of the 16S rDNA amplicons by limited cycle PCR. Sequencing libraries were validated using a DNA chip for the Agilent 2100 Bioanalyzer (Agilent Technologies, Palo Alto, CA, USA) and quantified by Qubit and real-time PCR (Applied Biosystems, Carlsbad, CA, USA). DNA libraries were multiplexed and loaded on an Illumina MiSeq instrument according to manufacturer’s instructions (Illumina, San Diego, CA, USA). Sequencing was performed using a 2 × 250 paired-end (PE) configuration; image analysis and base calling were conducted by the MiSeq Control Software (MCS) on the MiSeq instrument. Initial taxonomy analysis was carried out on Illumina Basespace cloud computing platform.

Sequence data resulting from the sequencing process were processed using a proprietary analysis pipeline from GENEWIZ. The QIIME data analysis package was used for 16S rRNA data analysis. The forward and reverse reads were joined and assigned to samples based on barcode and truncated by cutting off the barcode and primer sequence. Quality filtering on joined sequences was performed and sequence which did not fulfill the following criteria were discarded: sequence length < 200 bp, no ambiguous bases, mean quality score ≥ 20. Then, the sequences were compared with the reference database (RDP Gold database) using the UCHIME algorithm to detect chimeric sequence, and then the chimeric sequences were removed. The effective sequences were used in the final analysis. Sequences were grouped into operational taxonomic units (OTUs) using the clustering program VSEARCH (1.9.6) against the Silva 119 database pre-clustered at 97% sequence identity. The Ribosomal Database Program (RDP) classifier was used to assign taxonomic category to all OTUs at confidence threshold of 0.8. The RDP classifier uses the Silva 119 database which has taxonomic categories predicted to the species level. Sequences were rarefied prior to calculation of alpha and beta diversity statistics. Alpha diversity indexes were calculated in QIIME (1.9.1) from rarefied samples using for diversity the Shannon index, for richness the Chao1 index. β-diversity measures (bar charts, Bray–Curtis (BC) dissimilarity) were generated using R (R version 3.6.2, Vienna, Austria) and the package Vegan (Ecology Package. R package version 2.5–6. https://CRAN.R-project.org/package=vegan, accessed on 11 November 2020). Statistical analysis (Student *t*-test, Kruskal-Wallis, ANOVA, PERMANOVA) was performed in R (R version 3.6.2) and ToxRat (version 3.3.0, ToxRat Solutions GmbH, Alsdorf, Germany). For multiple testing, *p* < 0.05 was considered to be statistically significant, and for the Student *t*-test a one-sided analysis was performed.

### 2.5. Gold Analysis

The Au concentration in activated sludge was measured, carrying out an aqua regia extraction following the guideline DIN EN 16174:2012-11 [19] with an open extraction under reflux. Half a gram of the dried and finely ground sample was transferred to a decomposition vessel and moistened with two drops of water; 6 ± 0.1 mL 37% hydrochloric acid, 7.4 ± 0.1 mL 30% hydrochloric acid, and 2 ± 0.1 mL nitric acid were added in succession and mixed well. The temperature of the extraction mixture was increased to 175 ± 5 °C at a rate of about 10 °C/min to 15 °C/min and held at 175 ± 5 °C for 10 ± 1 min before allowing the vessel to cool to room temperature. After cooling, the extract was transferred to a 25 mL volumetric flask and then filled up to 25 mL with ultrapure water. If necessary, the extract was filtered through a 0.2 µm membrane filter to remove insoluble residues.

The Au concentrations were measured in the digested samples via ICP-MS (Agilent 7700 ICP-MS, Agilent Technologies Germany GmbH, Waldbronn, Germany) and ICP-MS/MS (Agilent 8900 ICP-MS/MS; Agilent Technologies) using the isotope 197Au in different gas modi (7700 ICP-MS: noGas, Helium and HiHelium; 8900 ICP-MS/MS: noGas, Oxygen and NH_3_). Each measurement series was verified by the use of quality control standards (certified standard not used for calibration) as well as recalibration samples.

## 3. Results

### 3.1. General Changes of Microbial Diversity

The mean number of sequences per eDNA sample ranged from 98,436 to 113,318 across all samples analyzed (Supplementary Materials Table S1). Data are accessible at NCBI (BioProject accession number PRJNA699734). The high number of sequences observed enabled the identification of microorganisms at different taxonomy levels per sample. The composition and diversity of the sludge were assessed during the complete tests, from collection of fresh sludge (day −8) until completion of the experiment (day 9). The diversity within a community (alpha-diversity) was described using the number of observed and predicted (Chao1) OTUs that characterize the richness of the microbiomes, and the Shannon and Simpson indexes which reflect the equivalence among microbial communities from the different samples (Supplementary Table S2).

From the collection of fresh sludge until the test start (first addition of AuNPs or dispersant), in both experiments the Chao 1 index did not vary significantly (Kruskal-Wallis, *p* < 0.05), while Shannon and Simpson indices varied significantly within each treatment over time (Kruskal-Wallis, *p* < 0.05). However, between days 3 and 9 there is no significant difference between the three indices, including controls and dispersant (Kruskal-Wallis, *p* > 0.05).

Based on the Simpson index, a high diversity was observed in the sludge from collection until the test end (experiment 1: 0.98–0.99; experiment 2: 0.95–0.98) (Supplementary Table S2). The percentage of total species represented in a sample (Good’s coverage) ranged from 99 to 100% in all samples, highlighting that the majority of the microbial community was captured. The high values of the Shannon index (experiment 1: 6.81–7.51; experiment 2: 5.58–6.15) indicated that the sample possessed rich communities which were not dominated by a few abundant taxa.

**Figure 2:**
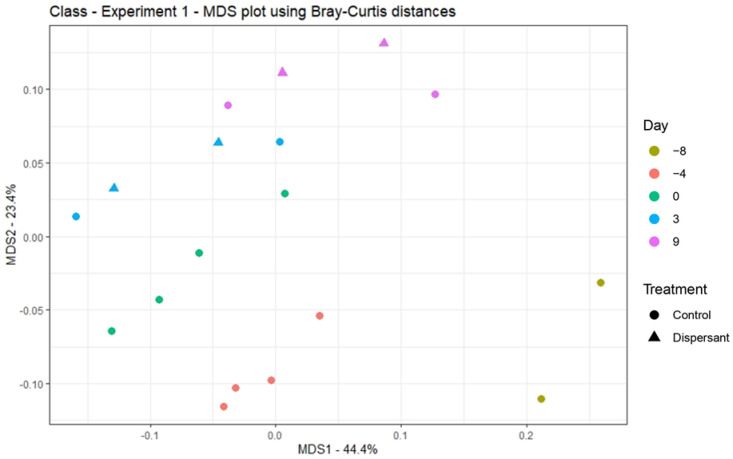
Principal coordinate analysis (PCoA) of bacterial communities, evaluated to class level, using Bray–Curtis distance. Symbols represent control (●) and dispersant (Δ), and color codes represent the different sampling points: yellow (day −8), red (day −4), green (day 0), blue (day 3), and purple (day 9).

Principal coordinate analysis (PCoA), displaying dissimilarities among communities (using BC distances), was performed at class level to visualize the differences among sampling times and the dispersant effect (Figure 2). No clustering is observed for the treatments used. Here, the larger distance was found between samples from the fresh sludge (day −8) until the test start (day 0), but not between the treatments used (control and dispersant), indicating that the application of dispersant did not significantly affect the diversity (*p* > 0.05). The PCoA analysis conducted to genus level (Supplementary Figure S1) showed the same trends as observed at the class level.

**Figure 3:**
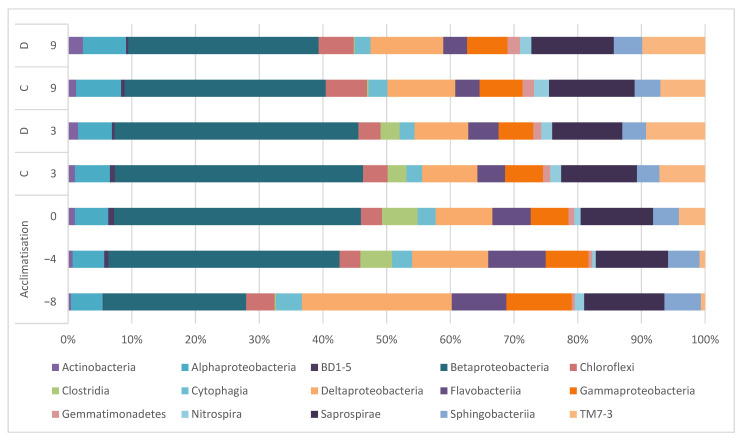
Mean abundances at class level of the sludge community from collection of fresh sludge until day 9 (C = control Table 7. increased their relative abundance over time. No changes in relative abundance were observed for classes such as Saprospirae, Sphingobacteria, or the nitrite oxidizing *Nitrospira*. For all described classes, no significant difference (Student *t*-test, *p* > 0.05) was observed between control and dispersant (evaluated at days 3 and 9). The analysis conducted to genus level (Supplementary Figure S2) confirmed that there are no significant differences (Student *t*-test, *p* > 0.05) between control and dispersant.

Univariate analysis of the bacterial composition of the treatment plants are illustrated at the class level (Figure 3). Significant differences (PERMANOVA, *p* < 0.05) in composition were detected between the fresh sludge (day −8) and the sludge at test start (day 0). Thereafter, the changes at the class level are considered to be nonsignificant (PERMANOVA, *p* > 0.05). While there are small changes over time, the differences between the control and the set-up receiving dispersant (day 3 and day 9) seem to be even smaller indicating that the most influence is caused by the addition of synthetic sewage and not by the dispersant.

### 3.2. Au Analysis in Activated Sludge

At test termination, samples were taken from activated sludge and effluent from the STP to determine the fate of the AuNPs in the STP. The results show a high variability between the two replicates. While in replicate 1 the recovery of Au in activated sludge was at 90%, it was at 39.2% in replicate 2. However, the measured Au concentrations in the effluent of the STP was comparable in both treatments at 0.6% of the total added Au. The results are compiled in Table 2.

### 3.3. Effect of AuNPs on Microbial Population

**Figure 4:**
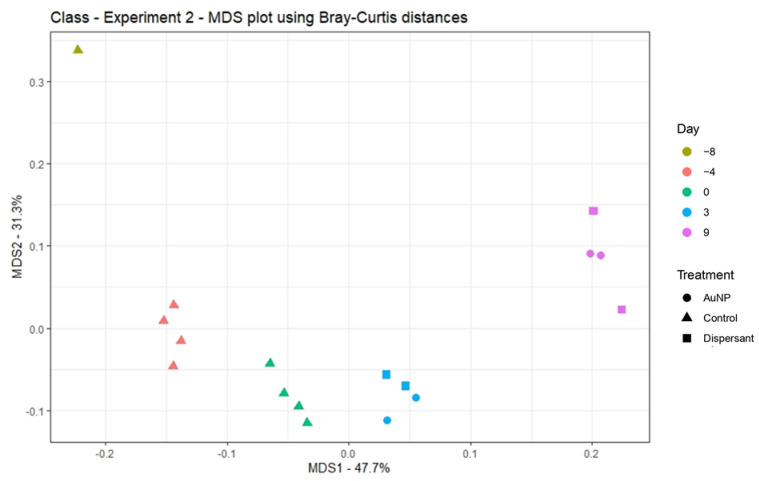
PCoA of bacterial communities, evaluated to class level, using Bray–Curtis distance. Scheme 8: Red (day −4), green (day 0), blue (day 3), and purple (day 9).

The mean number of sequences per eDNA sample ranged from 27,144 to 70,434 across all samples analyzed (Supplementary Table S3). The high number of sequences observed enabled the identification of microorganisms at different taxonomy levels per sample. Data are accessible at NCBI (BioProject accession number: PRJNA699745). Principal coordinate analysis (PCoA), displaying dissimilarities among communities (using BC distance), was performed to visualize the differences among treatments and sampling times (Figure 4). The sample from fresh sludge (day −8, yellow) showed the greatest divergence from the other samples, as observed also in experiment 1. No clustering is observed for the treatments (AuNPs or dispersant) applied, indicating that neither the application of dispersant nor AuNPs had a significant effect in the community (PERMANOVA, *p* > 0.05). The PCoA analysis conducted to genus level (Supplementary Figure S3) showed the same trends observed at class level.

**Figure 5:**
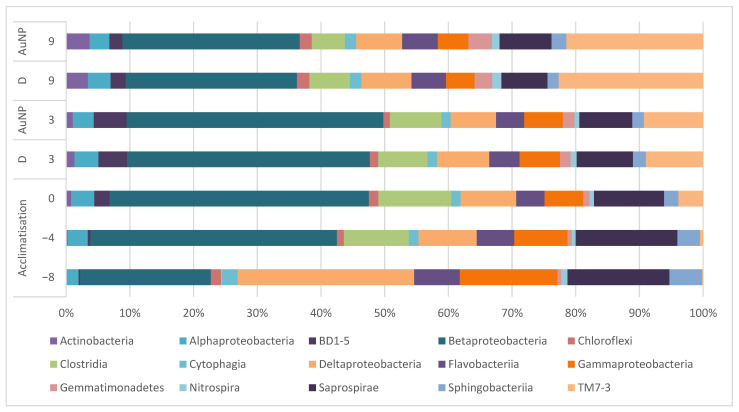
Mean abundance at class level of the sludge: during the experiment course, and before and after the application of dispersant (D) and AuNPs.

Univariate analysis of the bacterial composition of the treatment plants is illustrated at the class level (Figure 5). Differences in the relative abundance were observed during the experiment (from day −8 to day 9). However, the changes in composition were not significantly influenced by the treatments with the dispersant (experiment 1) or the AuNPs (experiment 2) (Student *t*-test evaluated at day 3 and 9, *p* > 0.05). This is also confirmed with the analysis to genus level (Supplementary Figure S4).

Similar to the first experiment, the descriptive evaluation of the data indicates that Actinobacteria, Clostridia, and TM7-3 were not detected in fresh sludge, but after the addition of synthetic sewage they were detected, and Actinobacteria and TM7-3 increased their relative abundance over time. The relative abundance of other species, e.g., *Flavobacteria*, remained unchanged with time and application of dispersant or AuNPs (Student *t*-test evaluated at day 3 and 9, *p* > 0.05).

### 3.4. Effect of Dispersant and AuNPs on the STP Function

In the first experiment, the effect of the dispersant compared to the STP only fed with synthetic sewage (control) was investigated. The results revealed that there was no impact on parameters like nitrate, nitrite, and ammonium (Table 3) nor on DOC elimination (Supplementary Table S4) in the effluent of the STP and therefore on the function of the STP. Neither the denitrification nor nitrification appeared to be influenced by the dispersant. The DOC elimination was constant and above 95% in all replicates. The development of pH and dry matter content over time was comparable.

Based on these results, in the second experiment with a comparable test setup, the effect of AuNPs on the function of the STP was investigated. The dispersant was used as a control. Here, no impact on the DOC elimination (Supplementary Table S4), the development of pH (Supplementary Table S5), or the dry matter content over time was observed.

In addition, based on the measured ammonium values in the effluent, the first step of the nitrification appeared not to be affected by the AuNPs addition over 10 days. There was no statistically significant difference between the ammonium concentrations measured in the effluent containing dispersant and the effluent of the AuNPs treatment (Table 3). However, nitrite concentrations were significantly lower in the AuNP treatment. In addition, the difference between the nitrite concentrations in the effluent of the two treatments became more significant with increasing test duration.

## 4. Discussion

### 4.1. General Change of Microbial Diversity over Time

One objective of the present study was to show whether a representative microbial diversity remains under standardized test conditions as described in OECD 303A using synthetic sewage as influent. Full-length sequencing of the 16S rRNA gene has been a widely used tool, which provides reliable taxonomic information, in the best case at the species level. In our study, we have used this technique to evaluate the general bacterial composition of a simulated sewage treatment plant and its changes over time due to the application of AuNPs and dispersant. The results indicated that first, changes occurred in terms of richness but not in terms of effective diversities. Second, the changes in the population occurred principally during the acclimatization phase, but once the addition of the test substances (AuNPs or dispersant) started, the community was already established and remained constant without significant changes.

The bacteria composition in the present study, evaluated changes at a class level, revealed commonly described environmental bacteria. Proteobacteria (β, α, δ) accounted for more than 50%. Proteobacteria have been recognized as key constituents in both industrial and municipal sewage treatment plants [20,21,22,23]. Juretschko et al. [24] observed the composition of the microbial community in activated sludge of an industrial STP. Of the 94 almost full-length 16 sRNA gene clones they derived, 59% were associated with the Proteobacteria (β, α, δ) followed by green non-sulfur bacteria and Planctomycetes. Additionally, they found Verrucomicrobia, Acidobacteria, *Nitrospira*, Bacteroidetes, Firmicutes, and Actinobacteria. In total, in our experiments a highly diverse microbial community structure was also indicated by several indices such as the Simpson Index (0.95–0.98). For example, we found *Nitrospira*, but also Nitrosomonadaceae, as nitrifying bacteria. Nitrification is an important process in STPs which is accomplished by nitrifying bacteria. Both microbial groups have been widely identified in sludge, using different techniques [23,24,25,26,27,28].

The general composition of the fresh sludge collected in two sampling periods (summer and winter) did not show changes in the general microbial composition found, and only minor changes in the bacterial composition regarding the relative abundance. This minor seasonal changes in sludge have been described already by Winkler et al. [29], indicating that at several determinations throughout a period of 400 days, the microbial populations of the activated sludge moved closely around the initial population. However, investigations of Zhang et al. [30] showed that seasonal changes could be more significant depending on the different wastewater processing systems.

The greatest differences in the microbial community structure of individual samples were found right at the beginning of the studies. Here, we observed significant changes in the microbial composition between the fresh activated sludge collected from the municipal sewage treatment plant and the activated sludge fed with synthetic sewage afterwards. Changes in the bacterial community of WWTPs can be affected by several factors such as the composition of the wastewater [31,32,33], the temperature [34], gas/water ratio [34,35], and hydraulic retention time [32]. In the present study a controlled simulated treatment plant was used, in which those factors remained stable during the experiment and can be excluded as the reason for the observed changes. The addition of synthetic sewage to feed the activated sludge introduced changes in the relative abundance of some classes, e.g., β-, δ-, and γ-Proteobacteria. However, our experiment was still characterized by a high degree of uniformity (Chao1 index), which has been previously discussed as an indicator of resilience against selective stress [36].

Modifications in the microbial community structure can result in modifications of the activated sludge floccules [37]. This must be kept in mind when the focus is on adsorption of NPs and the consequences for ecotoxicity.

The OECD test guideline 303 used in the present study describes a higher tier approach to assess the degradability of a test substance. The composition of the synthetic sewage was developed to maintain sufficient degradation capability of the activated sludge, but also to simulate a kind of worst case without any microbial specialists for the degradation of specific substances. As outlined above, one the main reasons for the significant change of the microbial structure in the first days of the laboratory experiment is the composition of the synthetic sewage which differs from municipal wastewater. Yang et al. [31] analyzed the composition of industrial and municipal wastewater and determined low concentrations of phenol, formaladehyde, aniline, surfactants, and chlorobenzene in the municipal wastewater. This indicates a much higher diversity of carbon sources compared to the synthetic sewage which only contains peptone, meat extract, and urea which can affect the composition of the microbial community.

### 4.2. Fate of AuNPs in the STP

Due to their use in medical products and other fields, AuNPs will enter the environment and ultimately end up in the STP. The fact that most metal or metal oxide nanoparticles sorb to activated sludge has already been shown in several publications [38,39,40,41,42]. The results of the actual study are no exception. In the two replicates, 39.2% (replicate 2) and 90% (replicate 1) of Au were found in the sludge while only a low Au concentration was found in the effluent. Part of the sludge from each replicate was used for further studies on its effects in soil (Hund-Rinke et al. in preparation). After the sludge was mixed with the soil, the Au content was measured again. Both replicates showed a comparable proportion of Au in soil. While for replicate 1 the recovery was 90% in the sludge, the recovery in soil was 93%. The high retention of AuNPs in sludge is confirmed by the recovery of replicate 2 in soil (131%). This indicates that the low recovery of 39.2% in the activated sludge of replicate 2 can be explained by an uneven distribution of AuNPs in the activated sludge resulting in a high variability of Au in sludge. Nevertheless, the results of the study show that a large proportion of AuNPs sorb to activated sludge and can ultimately end up via the sludge on agricultural land, which in turn pose a risk to terrestrial organisms and the structural diversity of microorganisms in soil.

It is important to put the concentrations used in the experiment and their results in relation to environmental concentrations. Measured concentrations cover the Au release of all uses into the environment, but are characteristic for specific locations and scenarios, whereas modeled concentrations describe the release more generically. For Au, only modeled environmental concentrations were found in the currently available literature. For the UK and US, the mean annual predicted environmental concentrations of medical AuNPs in activated sludge were estimated at 124 and 145 μg/kg, respectively [18]. The concentration in the activated sludge, about 7 g/kg, used for the experiments exceeded the PEC value by a factor of 10,000 and represents a worst-case scenario. If effects are not observed at such high concentrations, unacceptable effects in the environment are considered unlikely.

### 4.3. Effect of AuNPs on Structural Diversity and Function of a STP

In the present study, it was investigated whether only considering the functional properties of a STP, such as DOC elimination, is sufficient for an assessment of AuNPs or whether the influence on microbial diversity also needs to be considered. The results can provide a significant input for the interpretation of results from the regulatory point of view.

STPs are a complex system to analyze, and many factors can alter the results. Nitrification is a multistep process and drives nutrient removal in STPs to keep the needed ecological balance. Ammonium-oxidizing bacteria transform ammonium to nitrite and nitrite-oxidizing bacteria convert nitrite into nitrate. This aerobic process is driven by oxygen, and often the microorganisms must compete for it and other nutrients with faster-growing heterotrophs [43].

According to the best of our knowledge, there is a gap in the literature regarding the effect of AuNP on the microbial composition of activated sludge especially in environmental realistic scenarios such as a sewage treatment plant simulation. In our experiment, no changes in the bacterial composition and the diversity were detected by the added AuNPs in a high concentration.

In addition, we observed no inhibitory effect on the degradation of the added carbon sources and on the nitrification by the added AuNPs. This is in-line with results of Luo et al. [15] and García et al. [16], who reported no inhibition of the AOB biodiversity, abundance, and ammonium oxidation. However, we found that the nitrite concentrations were significantly lower in the AuNP treatment compared to the setup with dispersant, which could not be explained by a higher standard deviation as, e.g., for nitrate. The difference became even more significant with increasing test duration. Compared to the results of the first experiment and to the AuNP treatment, the nitrite concentrations in the setup with the dispersant of the second experiment were comparable high. Therefore, the difference between the AuNP and dispersant setup should not be overestimated and considered as stimulation of the nitrite oxidation by AuNPs. As far as we know, there is no evidence of AuNPs promoting nitrification in the current literature. Instead, there is rather evidence of an inhibitory effect or a lack of effect.

Although 5 nm AuNP had no effect on the growth of *E. coli*, a cell–AuNP interaction was observed resulting in an increased cell length by a factor of 8 and a tendency for incorporation within bacterial cells [44]. The penetration of the cell wall by an AuNP (25 ± 5 nm) was also demonstrated for *Corynebacterium pseudotuberculosis* [45], whereas a 30 nm-AuNP only showed strong adherence to a variety of gram− and gram+ bacteria with a relationship to the bacterial zeta potential [46]. Singh et al. [47] observed no effect on growth of *Pseudomonas aeruginosa* and *Escherichia coli* by AuNPs (∅ 12 to 18 nm) even at the highest test concentration of 200 µg/mL. However, an inhibition of their biofilm production at a concentration of 12.5 µg/mL was observed. Non-lethal damage or inhibitory effect on expression of genes related to motility and biofilm formation could be potential influencing factors [48,49]. A biofilm composition of only one bacterium has no environmental relevance and can differ significantly from a real composition. This can be the reason for the different observations in our experiments where we detected no effects on the determined functions or on the composition of the microbial structure or the formation of bulking sludge by the AuNPs. Only 5 to 20% of the organic material of activated sludge are bacteria. The remaining portion can be regarded as dead material causing many of the colloidal-chemical properties of the floc [50] and can affect ecotoxicity. A review of 66 publications revealed a trend that higher natural organic matter (NOM) concentrations reduce ecotoxicity. Potential explanations are that NOM can hinder the nanomaterials coming into contact with the organisms and that it can form complexes with dissolved ions released by the NMs. Additionally, it can serve as nutrient source improving the resistance of the organisms [51].

## 5. Conclusions

Adequate functioning of a sewage treatment plant is essential to protect the downstream aquatic environment and to minimize operational costs [1]. The OECD 303A [2] was developed for the biodegradability testing of water-soluble and non-volatile chemicals. Now, it is additionally used to study the fate of nanoparticles and to receive activated sludge with adsorbed nanoparticles for further studies such as investigations on ecotoxicological effects in soil when activated sludge is used as fertilizer.

The main objective was to show whether a representative microbial diversity remains under standardized test conditions as described in OECD 303A [2] using synthetic sewage as influent. Furthermore, we investigated whether just considering the functional properties of a STP, such as DOC elimination, is sufficient for an assessment of AuNPs or whether the influence on microbial diversity also needs to be considered.

Our results show that if the aim of a study is to gather specific information on the adsorption of nanoparticles or if the sludge might be used for studies in soil on ecotoxicological effects by adsorbed pollutants, the development of a more realistic composition of the synthetic sewage is recommended minimizing the changes in the microbial community such as the reduction of the β- and γ-Proteobacteria and Saprospirae which we observed in our study with the synthetic sewage according to the OECD test guideline.

Focusing on a potential hazard assessment, even though gold has antibacterial properties, no adverse effects on the function and structure of the microorganisms in the sewage treatment plant could be detected at concentrations which increased the modeled PEC values by a factor of about 10,000.

Comparable to other metallic nanomaterials, gold nanomaterials also sorbed to a large extent to the activated sludge. If activated sludge is used as fertilizer on agricultural land, gold nanoparticles can be introduced into soils. In this case, the effect on soil (micro)organisms must be investigated more closely, also taking into account the structural diversity.

## Figures and Tables

**Table 1:** Dispersant and AuNP characterization (Colorobbia Consulting srl). Abbreviations: nm = nanometer; mV = millivolt; %wt = weight percentage.

Sample Name	Au Concentration [mg/mL]	Hydrodynamic Diameter [nm]	Zeta Potential [mV]	Appearance	pH
**Dispersant**	-	-	Not applicable	Transparent fluid	5.0
**AuNPs**	5 ± 0.5	25.2 ±0.6	−15.2 ± 1.0	Magenta fluid	5.0

**Table 2:** Fate of Au in the sewage treatment plant (STP): Measured gold concentration in activated sludge and effluent.

Au in Sewage Sludge	Replicate 1	Replicate 2	Mean
Nominal Au [mg/g dm sludge]	7.6	7.6	7.6
Measured Au [mg/g dm sludge]	6.8	3.0	4.9
Percent of added Au in sludge	90.0	39.2	64.6
Measured Au [mg/L effluent]	1.8	1.6	1.7
Percent of added Au in effluent	0.7	0.6	0.6

**Table 3:** Measured values of ammonium, nitrite, and nitrate in the effluent of the STPs in experiments 1 and 2. Student *t*-test. Alpha = 0.05; one sided smaller (nitrite) and one sided greater (nitrate). * *p* < 0.05.

Day	Experiment 1				Experiment 2			
	Control		Dispersant		Dispersant		Au-NP	
	NH_4_	SD	NH_4_	SD	NH_4_	SD	NH_4_	SD
	[mg/L]		[mg/L]		[mg/L]		[mg/L]	
−6	13.0	4.2	11.5	0.7	25.0	9.9	19.5	6.4
−4	6.7	8.9	0.4	0.0	5.6	6.2	3.4	3.7
0	0.3	0.3	0.2	0.1	0.5	0.2	0.5	0.4
3	0.5	0.4	0.3	0.1	0.6	0.4	0.5	0.1
6	0.2	0.1	0.1	0.0	0.2	0.1	0.7	0.4
9	0.1	0.0	0.4	0.4	0.3	0.0	0.5	0.2
**Day**	**NO_2_**	**SD**	**NO_2_**	**SD**	**NO_2_**	**SD**	**NO_2_**	**SD**
	**[mg/L]**		**[mg/L]**		**[mg/L]**		**[mg/L]**	
−6	6.0	0.3	9.5	1.3	8.0	1.8	5.0	2.1
−4	6.7	4.8	11.3	0.5	20.8	4.0	15.5	1.3
0	6.4	6.7	8.6	1.2	16.0	0.6	11.0	2.8
3	5.3	5.6	3.2	1.5	17.4	0.3	12.1	2.4
6	1.3	0.4	1.0	0.0	12.3	2.7	5.5 *	0.4
9	1.2	0.3	1.0	0.0	16.6	3.7	3.1 *	0.7
**Day**	**NO_3_**	**SD**	**NO_3_**	**SD**	**NO_3_**	**SD**	**NO_3_**	**SD**
	**[mg/L]**		**[mg/L]**		**[mg/L]**		**[mg/L]**	
−6	15.5	9.2	19.5	2.1	27.5	13.4	32.0	8.5
−4	18.5	14.8	22.0	5.7	24.5	10.6	32.0	11.3
0	26.5	9.2	31.0	1.4	31.5	12.0	43.0	22.6
3	27.5	9.2	44.5	0.7	29.0	5.7	46.5	19.1
6	20.5	2.1	24.5	0.7	30.0	5.7	50.5	14.8
9	25.0	2.8	21.0	2.8	32.0	4.2	54 *	9.9

Note: Measurements started at day −6 once the sludge was added into the STP (day −8) and the system had some time to settle. The measurements during the acclimatization phase are only to show when a stable system is achieved.

## Data Availability

The datasets generated during and/or analysed during the current study are available from the corresponding author on reasonable request. Metagenomic data are accessible at NCBI (BioProject accession number PRJNA699734 and PRJNA699745).

## Supplementary Materials

The following are available online at https://www.mdpi.com/2305-6304/9/3/54/s1. Table S1. Number of sequences obtained and quality scores of the sequences for the 1st experiment (control/dispersant experiment). Table S2. Alpha diversity index of bacterial microbiomes in the 1st experiment (a) and 2nd experiment (b). Table S3. Number of sequences obtained and quality scores of the sequences for the 2nd experiment (dispersant and AuNPs experiment). Table S4. Measured dissolved organic carbon concentration in influent and effluent of the STPs and the elimination rate throughout the two experiments. Table S5. Measured pH values in the denitrification, nitrification and settling tank of the STPs throughout the two experiments. Figure S1. PCoA of bacterial communities, evaluated to genus level, using Bray-Curtis distance. Symbols represent control (●) and dispersant (Δ), and color codes represent the different sampling points: yellow (day −8), red (day −4), green (day 0), blue (day 3) and purple (day 9). Figure S2. Mean abundances at genus level of the sludge community from collection of fresh sludge until day 9 (C = control treatment and D = dispersant treatment). Figure S3. PCoA of bacterial communities, evaluated to genus level, using Bray-Curtis distance. Symbols represent control (Δ), applied with AuNPs (●) and dispersant (▪), and color codes represent the different sampling points: yellow (day −8), red (day −4), green (day 0), blue (day 3) and purple (day 9). Figure S4. Mean abundance at genus level of the sludge, during the experiment course, before and after the application of dispersant (D) and AuNPs.
