# Supplementary material for: Microbial Population Dynamics in Model Sewage Treatment Plants and the Fate and Effect of Gold Nanoparticles
Source: Toxics. 2021 Mar 10;9(3):54. doi: 10.3390/toxics9030054 (PMC8001127; doi:10.3390/toxics9030054)
Supplement: Supplementary file 1 [file toxics-09-00054-s001.pdf]

toxics

MDPI

Article

Supplementary Material: Microbial Population Dynamics in Model Sewage Treatment Plants and the Fate and Effect of Gold Nanoparticles

Karsten Schlich, Cecilia Díaz, Benjamin Gomez Pizarro, Burkhard Knopf, Ruben Schlinkert, Franziska Frederike Wege, Anne Jurack and Kerstin Hund-Rinke

Table S1. Number of sequences obtained and quality scores of the sequences for the 1st experiment (control/dispersant experiment).

GENEWIZ NGS DATA REPORT							
**Sample**	**Length (bp)**	**# Reads**	**Bases (bp)**	**Q20 (%)**	**Q30 (%)**	**GC (%)**	**N (ppm)**
1	249.52	98,436	24,561,461	97.28	95.89	53.28	1.42
2	249.57	97,808	24,410,236	97.13	95.59	54.20	2.54
3	249.53	108,208	27,001,593	93.27	90.48	53.16	2.22
4	249.55	113,318	28,278,197	93.34	90.56	53.17	0.00
5	249.53	103,112	25,729,944	93.26	90.47	53.23	0.00
6	249.53	104,504	26,077,044	92.97	90.05	53.30	0.00
7	249.54	100,426	25,060,715	93.36	90.61	53.10	1.60
8	249.53	102,456	25,565,843	92.96	90.03	53.23	2.97
9	249.54	100,540	25,088,593	93.18	90.33	53.27	0.20
10	249.53	104,496	26,074,971	93.19	90.33	53.52	0.69
11	249.55	106,570	26,594,453	96.91	95.37	53.36	0.75
12	249.56	109,128	27,234,016	96.87	95.32	53.43	0.18
13	249.54	105,718	26,381,097	96.95	95.43	53.26	0.00
14	249.56	111,004	27,701,636	96.80	95.18	53.70	0.00
15	249.57	106,562	26,594,728	96.93	95.38	53.42	0.83
16	249.52	101,180	25,246,477	96.76	95.13	53.52	3.56
17	249.57	103,002	25,706,130	96.88	95.34	53.41	0.00
18	249.56	109,834	27,410,112	97.01	95.51	53.43	0.22
19	249.55	112,472	28,067,249	96.63	94.96	54.20	1.50
20	249.61	110,260	27,521,466	96.68	95.05	53.59	0.00
21	249.57	110,580	27,597,078	96.76	95.15	53.86	1.38

Table S2. Alpha diversity index of bacterial microbiomes in the 1st experiment (a) and 2nd experiment (b).

	Day	Sample Type	Ace	Chao1	Shannon	Simpson	Goods_Coverage
a	−8	Control	496.15	498.50	7.51	0.99	1.00
	−4	Control	505.37	505.77	7.28	0.98	1.00
		Control	507.24	505.67	7.28	0.98	1.00
		Control	514.84	524.25	7.33	0.99	1.00
		Control	500.98	502.56	7.31	0.99	1.00
	0	Control	507.01	510.04	7.06	0.98	1.00
		Control	496.80	497.96	7.09	0.98	1.00
		Control	503.19	505.16	7.17	0.98	1.00
		Control	500.64	503.07	7.18	0.98	1.00
	3	Control	491.18	501.07	6.88	0.98	1.00
		Control	485.94	486.12	6.86	0.98	1.00
		Dispersant	497.92	498.92	6.95	0.98	1.00
		Dispersant	511.50	516.44	7.00	0.98	1.00
	9	Control	479.29	482.00	6.84	0.98	1.00
		Control	485.43	487.10	6.81	0.98	1.00
		Dispersant	487.70	499.46	6.93	0.98	1.00
		Dispersant	499.36	505.77	7.01	0.98	1.00
	Day	Sample Type	Ace	Chao1	Shannon	Simpson	Goods_Coverage
b	−8	Control	142.72	143.00	6.13	0.98	1.00
	−4	Control	147.96	147.50	6.15	0.98	1.00
		Control	145.50	146.00	6.08	0.98	1.00
		Control	147.54	147.67	6.05	0.97	1.00
		Control	145.45	144.75	6.13	0.98	1.00
	0	Control	148.99	151.00	6.08	0.98	1.00
		Control	152.65	155.00	6.03	0.97	1.00
		Control	151.21	151.00	6.08	0.98	1.00
		Control	151.42	152.50	6.03	0.97	1.00
	3	Dispersant	153.36	153.25	5.97	0.97	1.00
		Dispersant	148.79	148.62	5.88	0.97	1.00
		Au-NPs	154.56	154.62	5.95	0.97	1.00
		Au-NPs	151.37	151.62	5.83	0.97	1.00
	9	Dispersant	145.93	147.11	5.58	0.96	1.00
		Dispersant	143.94	145.67	5.61	0.95	1.00
		Au-NPs	152.35	152.00	5.62	0.95	1.00
		Au-NPs	148.82	147.50	5.75	0.96	1.00

Table S3. Number of sequences obtained and quality scores of the sequences for the 2nd experiment (dispersant and AuNPs experiment).

GENEWIZ NGS 16S-EZ ANALYSIS REPORT							
**Sample**	**Length (bp)**	**# Reads**	**Bases (bp)**	**Q20 (%)**	**Q30 (%)**	**GC (%)**	**N (ppm)**
FW01-1-16S	249.48	70,434	17,571,669	96.36	94.86	53.17	0.57
FW02-2-16S	249.43	33,228	8,288,110	94.29	92.20	53.17	0.48
FW03-3-16S	249.49	63,492	15,840,926	96.27	94.72	53.13	0.57
FW04-4-16S	249.46	49,244	12,284,184	96.12	94.51	53.09	0.00
FW05-5-16S	249.48	60,292	15,041,497	96.24	94.65	53.10	6.12
FW06-6-16S	249.54	58,266	14,539,872	96.49	94.98	53.45	0.14
FW07-7-16S	249.52	33,598	8,383,422	96.40	94.87	53.63	0.36
FW08-8-16S	249.56	63,900	15,946,803	95.87	94.17	53.36	1.25
FW09-9-16S	249.50	51,558	12,863,469	96.92	95.58	53.37	1.32
FW10-10-16S	249.43	28,894	7,207,084	95.05	93.20	53.45	0.00
FW11-11-16S	249.49	55,262	13,787,192	96.86	95.50	53.58	0.07
FW12-12-16S	249.51	38,796	9,679,822	96.94	95.60	53.44	1.65
FW13-13-16S	249.52	52,862	13,190,057	96.93	95.58	53.51	1.59
FW14-14-16S	249.45	44,378	11,070,042	97.12	95.87	53.41	1.81
FW15-15-16S	249.55	27,144	6,773,743	97.01	95.70	53.46	0.30
FW16-16-16S	249.52	49,148	12,263,374	96.57	95.14	53.46	1.30
FW17-17-16S	249.47	63,582	15,861,860	96.56	95.10	53.39	2.71

Table S4. Measured dissolved organic carbon concentration in influent and effluent of the STPs and the elimination rate throughout the two experiments.

Day	Experiment 1 (Control vs. Dispersant)					
	Control—Replicate 1			Control—Replicate 2		
	Influent [mg/L]	Effluent [mg/L]	DOC-Elimination [%]	Influent [mg/L]	Effluent [mg/L]	DOC-Elimination [%]
−6	175	6.4	96	143	6.8	95
–4	148	7.9	95	125	5.4	96
0	139	4.8	97	122	3.7	97
2	136	4.8	96	126	3.9	97
4	132	4.7	96	124	3.7	97
6	128	4.5	96	121	3.4	97
9	165	4.0	98	142	3.6	97
	Dispersant—Replicate 1			Dispersant— - Replicate 2		
Day	Influent [mg/L]	Effluent [mg/L]	DOC-Elimination [%]	Influent [mg/L]	Effluent [mg/L]	DOC-Elimination [%]
−6	146	6.8	95	151	6.5	96
–4	117	5.4	95	134	6.1	95
0	123	3.2	97	161	3.7	98
2	116	3.4	97	130	4.5	97
4	118	3.9	97	123	4.0	97
6	121	4.4	96	116	3.5	97
9	158	3.9	98	136	3.2	98
Day	Experiment 2 (Dispersant vs. AuNPs)					
	Dispersant—Replicate 1			Dispersant—Replicate 2		
	Influent [mg/L]	Effluent [mg/L]	DOC-Elimination [%]	Influent [mg/L]	Effluent [mg/L]	DOC-Elimination [%]
−6	151	5.9	96	154	4.0	97
−4	135	3.8	97	152	3.8	97
0	143	4.4	97	153	0.3	100
2	129	3.6	97	143	0.3	100
4	126	4.0	97	138	3.9	97
6	133	3.8	97	132	3.8	97
9	129	3.4	97	128	3.7	97
	AuNP—Replicate 1			AuNP—Replicate 2		
Day	Influent [mg/L]	Effluent [mg/L]	DOC-Elimination [%]	Influent [mg/L]	Effluent [mg/L]	DOC-Elimination [%]
−6	152	3.9	97	152	3.9	97
–4	147	4.4	97	149	3.9	97
0	145	4.3	97	148	4.1	97
2	137	4.2	97	149	4.1	97
4	160	4.1	97	145	3.8	97
6	117	3.9	97	135	3.8	97
9	117	3.4	97	119	3.5	97

Table S5. Measured pH values in the denitrification, nitrification and settling tank of the STPs throughout the two experiments.

Day	Experiment 1 (Control vs. Dispersant)					
	Control—Replicate 1			Control—Replicate 2		
	Denitrification	Nitrification	Settling Tank	Denitrification	Nitrification	Settling Tank
−6	7.5	7.5	7.3	7.5	7.5	7.4
−4	7.4	7.3	7.3	7.3	7.4	7.3
0	7.3	7.4	7.3	7.4	7.3	7.3
2	7.3	7.3	7.3	7.3	7.3	7.3
6	7.3	7.3	7.3	7.3	7.3	7.2
9	7.3	7.3	7.2	7.3	7.3	7.2
Day	Dispersant—Replicate 1			Dispersant—Replicate 2		
	Denitrification	Nitrification	Settling Tank	Denitrification	Nitrification	Settling Tank
−6	7.4	7.4	7.3	7.5	7.5	7.4
−4	7.4	7.4	7.3	7.4	7.3	7.3
0	7.3	7.4	7.3	7.3	7.3	7.4
2	7.3	7.4	7.3	7.3	7.3	7.3
6	7.4	7.4	7.3	7.3	7.3	7.2
9	7.3	7.3	7.3	7.3	7.3	7.2
Day	Experiment 2 (Dispersant vs. AuNPs)					
	Dispersant—Replicate 1			Dispersant—Replicate 2		
	Denitrification	Nitrification	Settling tank	Denitrification	Nitrification	Settling tank
−6	7.4	7.5	7.4	7.6	7.6	7.5
−4	7.3	7.2	7.2	7.4	7.3	7.1
0	7.2	7.0	7.0	7.3	7.0	7.0
2	7.2	7.0	7.0	7.3	7.0	7.0
6	7.3	7.1	7.1	7.3	7.1	7.1
9	7.2	7.0	6.9	7.2	7.1	7.0
Day	AuNP—Replicate 1			AuNP—Replicate 2		
	Denitrification	Nitrification	Settling tank	Denitrification	Nitrification	Settling tank
−6	7.5	7.3	7.3	7.5	7.3	7.2
−4	7.4	7.1	7.1	7.4	7.0	6.9
0	7.3	7.0	7.0	7.4	6.9	6.8
2	7.3	6.9	6.9	7.3	6.8	6.8
6	7.2	7.1	7.0	7.3	6.9	6.8
9	7.2	7.0	6.9	7.3	7.0	6.8

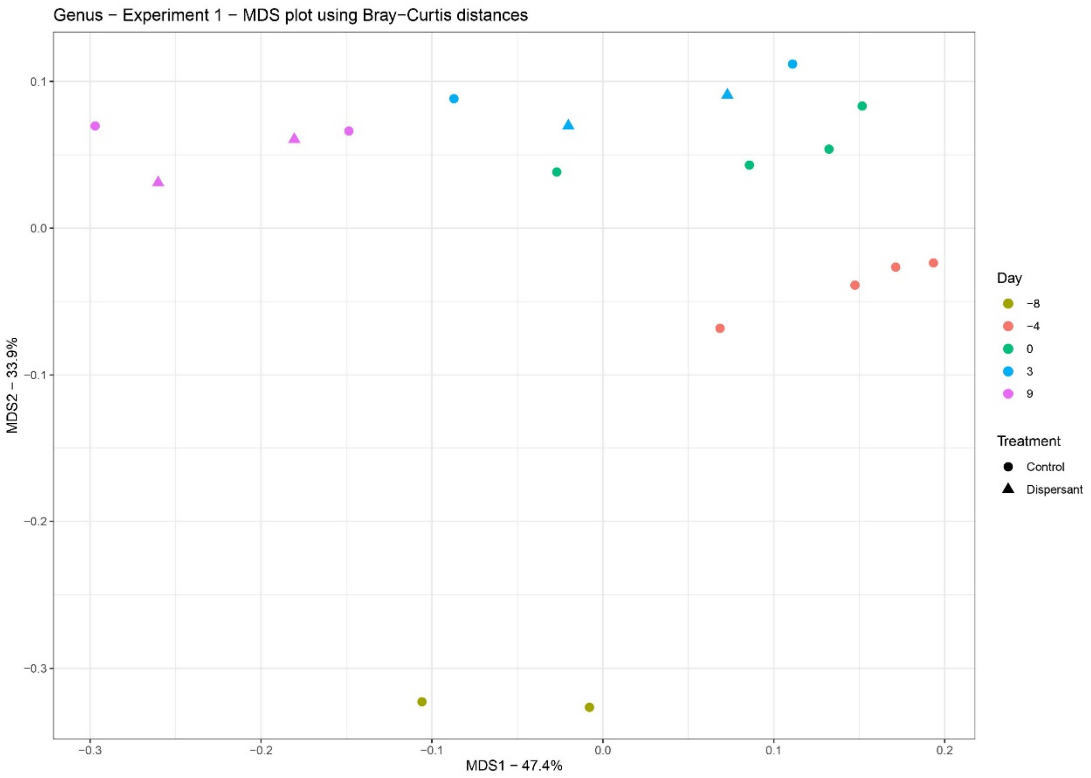

Figure S1. PCoA of bacterial communities, evaluated to genus level, using Bray-Curtis distance. Symbols represent control (●) and dispersant (Δ), and color codes represent the different sampling points: yellow (day −8), red (day −4), green (day 0), blue (day 3) and purple (day 9).

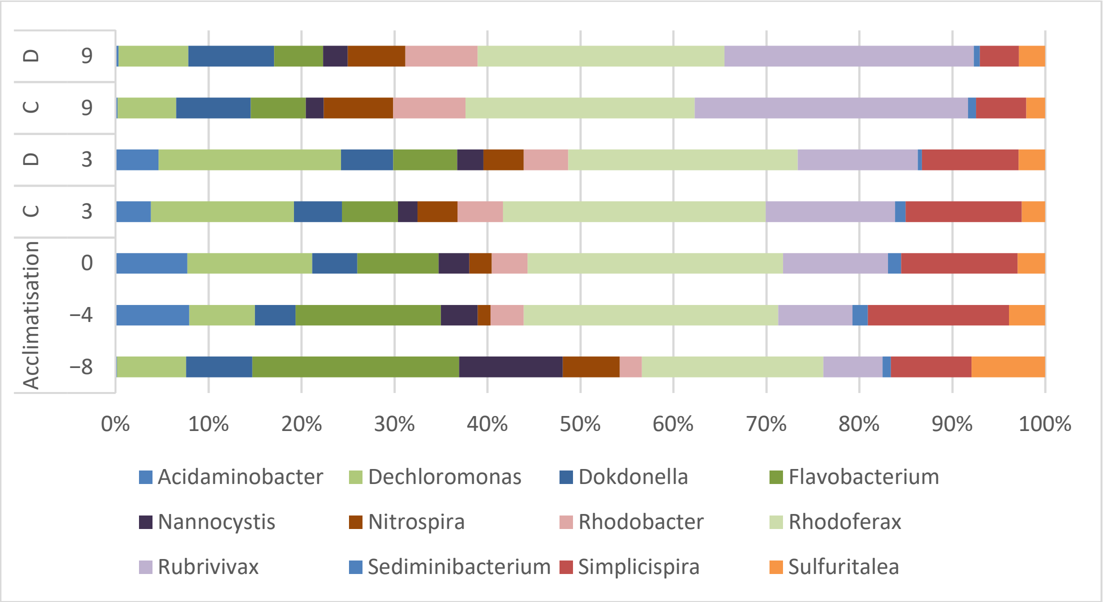

Figure S2. Mean abundances at genus level of the sludge community from collection of fresh sludge until day 9 (C = control treatment and D = dispersant treatment).

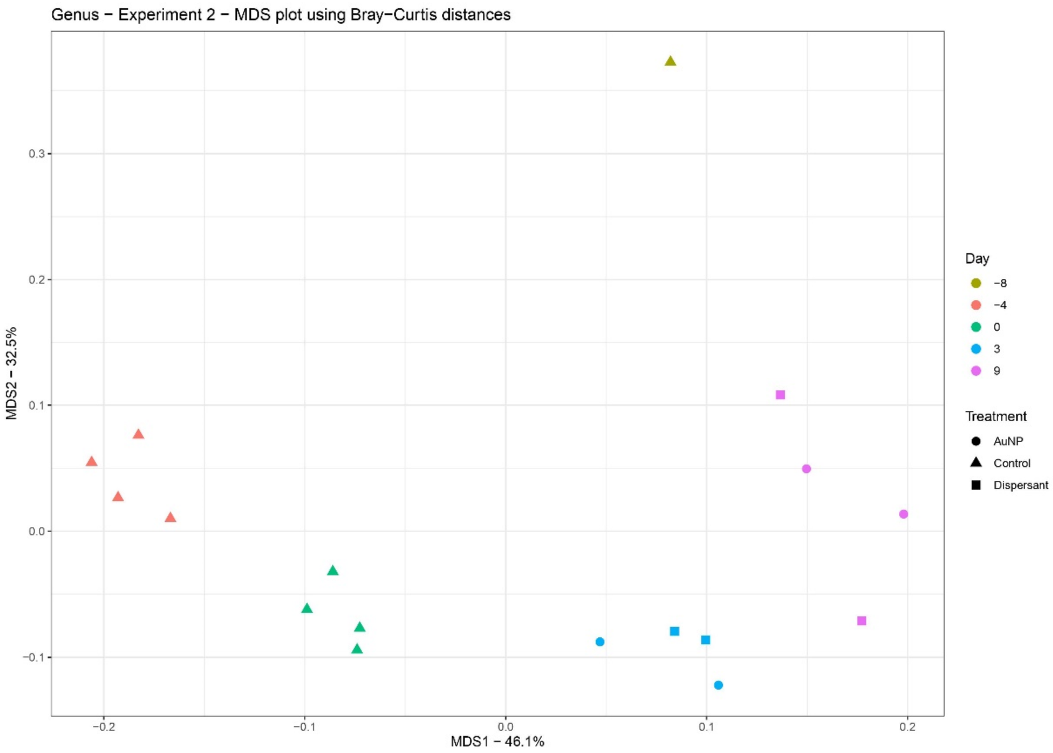

Figure S3. PCoA of bacterial communities, evaluated to genus level, using Bray-Curtis distance. Symbols represent control (Δ), applied with AuNPs (●) and dispersant (▪), and color codes represent the different sampling points: yellow (day −8), red (day −4), green (day 0), blue (day 3) and purple (day 9).

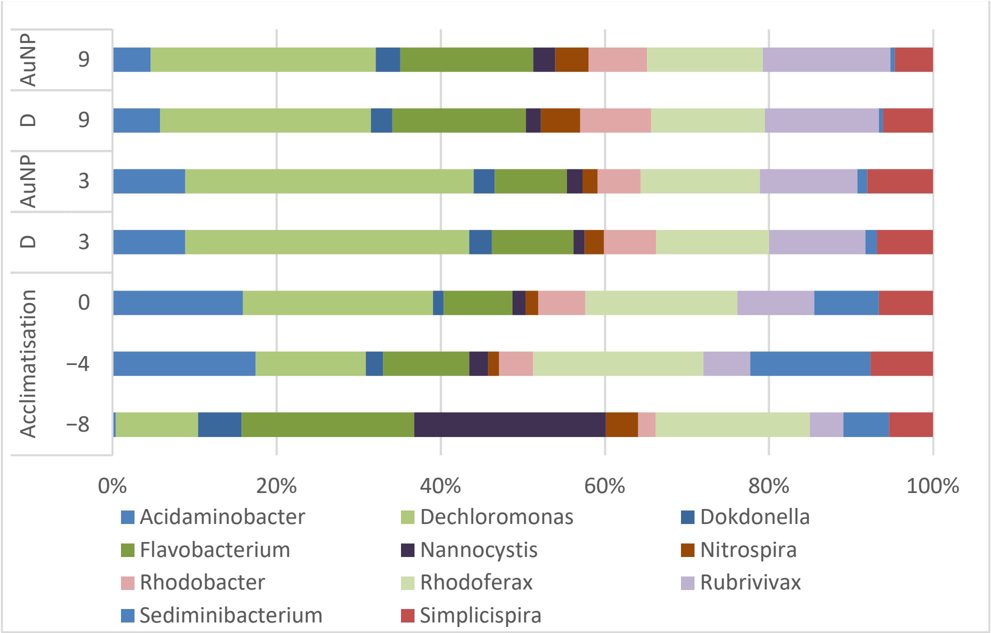

Figure S4. Mean abundance at genus level of the sludge, during the experiment course, before and after the application of dispersant (D) and AuNPs.
